# Thermal Adaptation of Conformational Dynamics in Ribonuclease H

**DOI:** 10.1371/journal.pcbi.1003218

**Published:** 2013-10-03

**Authors:** Kate A. Stafford, Paul Robustelli, Arthur G. Palmer

**Affiliations:** Department of Biochemistry and Molecular Biophysics, Columbia University, New York, New York, United States of America; Max Planck Institute for Biophysical Chemistry, Germany

## Abstract

The relationship between inherent internal conformational processes and enzymatic activity or thermodynamic stability of proteins has proven difficult to characterize. The study of homologous proteins with differing thermostabilities offers an especially useful approach for understanding the functional aspects of conformational dynamics. In particular, ribonuclease HI (RNase H), an 18 kD globular protein that hydrolyzes the RNA strand of RNA:DNA hybrid substrates, has been extensively studied by NMR spectroscopy to characterize the differences in dynamics between homologs from the mesophilic organism *E. coli* and the thermophilic organism *T. thermophilus*. Herein, molecular dynamics simulations are reported for five homologous RNase H proteins of varying thermostabilities and enzymatic activities from organisms of markedly different preferred growth temperatures. For the *E. coli* and *T. thermophilus* proteins, strong agreement is obtained between simulated and experimental values for NMR order parameters and for dynamically averaged chemical shifts, suggesting that these simulations can be a productive platform for predicting the effects of individual amino acid residues on dynamic behavior. Analyses of the simulations reveal that a single residue differentiates between two different and otherwise conserved dynamic processes in a region of the protein known to form part of the substrate-binding interface. Additional key residues within these two categories are identified through the temperature-dependence of these conformational processes.

## Author Summary

The relationship between enzymatic activity and protein stability has long been a difficult problem in the study of protein biochemistry. Enzymes may undergo structural changes in order to bind substrates, catalyze chemical reactions, and release products, but flexibility often is inversely correlated with thermodynamic stability. Proteins from organisms that are adapted to high temperature can be both more rigid and less active at ambient temperature than their homologs from organisms that grow at lower temperatures. For this reason, studying homologous pairs of proteins from organisms adapted to different thermal environments is a productive way to identify functionally important motions. In this work we perform comparative analyses of molecular dynamics simulations for five ribonuclease H proteins of varying thermal stabilities, isolated from organisms that grow in varying thermal environments. We identify two different mechanisms of motion in a region of the protein that interacts with substrate molecules, suggesting at least two forms of thermal adaptation in this protein family.

## Introduction

Structural changes are critical to the ability of proteins to execute biological function. Regions known to be in contact with substrates and to undergo conformational changes during the catalytic cycles of enzymes often are identifiable as particularly flexible by NMR spectroscopy [1], [2] and by computational methods [3]–[5]; however, mechanistic descriptions of the structural changes underlying flexibility are difficult to establish. Molecular dynamics (MD) simulations can complement observations made by NMR spectroscopy via direct simulation of functionally relevant dynamic processes [6]–[9].

The relationship between conformational dynamics and catalysis has been the subject of extensive recent debate [10], [11]. Although the majority of the controversy has focused on the question of whether dynamics have an effect on the chemical step in the catalytic cycle—and at best, the effect seems to be limited to fast-timescale, local motions [12]—questions remain regarding the role of dynamics in binding and orienting substrate and cofactors to generate the precise electrostatic preorganization thought to be required for catalysis [13], [14]. Thus larger-scale motions of enzymes, particularly in those regions known to interact with substrate, influence binding affinity, product release rates, and other processes relevant to determining the overall function of the enzyme.

Homologous pairs of proteins from mesophilic and thermophilic organisms have proven especially useful in understanding the functional aspects of protein dynamics [15]–[19]. Features thought to contribute to protein thermostabilization include more salt bridges, shorter loops, and better hydrophobic packing compared to proteins from mesophilic organisms [20], [21]. A number of examples have been identified in which a thermophilic enzyme is both more rigid and less active than its mesophilic homolog at ambient temperature [16], [22], [23], leading to the hypothesis that motions critical to function can be specifically identified by comparing the dynamics of such homologous pairs. The ribonuclease HI (RNase H) homologs from the mesophilic bacterium *Escherichia coli* (ecRNH) and the thermophilic bacterium *Thermus thermophilus* (ttRNH) form one such well-characterized pair [24]–[28]. RNase H proteins are well-conserved endonucleases that are found in all domains of life and sequence-agnostically cleave the RNA strand of an RNA-DNA duplex substrate in a divalent cation-dependent manner [24]. The ttRNH homolog, despite 52% sequence identity with ecRNH and less than 1 Å 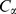 RMSD in secondary structural elements, has reduced enzymatic activity [25] and greater thermal stability [26], [27] compared to ecRNH. Reciprocal mutations have identified five distinct sites that collectively contribute about half of this stability difference [28]. More recently, similar analyses have identified mutations that confer increased thermostability to the homolog from the psychrotrophic bacterium *Shewanella oneidensis* (soRNH) [29]; like many proteins from cold-tolerant organisms [30], soRNH is natively thermolabile compared to its mesophilic homolog. Furthermore, comparison of the thermodynamic parameters of ecRNH, ttRNH, and an additional homolog from the moderately thermophilic bacterium *Chlorobium tepidum* reveals that the more thermostable proteins share a common mechanism of stabilization in the form of increased values of 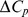, likely owing to the existence of residual structure in the unfolded state [31], [32]. The structural and kinetic properties of these RNase H homologs are summarized in Table 1 and 2, respectively.

**Table 1:** Properties of structurally characterized RNase H homologs.

Protein	Source organism	Classification	PDB ID	Res (Å)	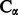 RMSD (Å)	% Seq ID	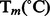
soRNH	*Shewanella oneidensis*	Psychrotroph	2E4L [55]	2.00	1.37	70%	53 [55]
ecRNH	*Escherichia coli*	Mesophile	2RN2 [56]	1.48	–	–	71 [55]
ctRNH	*Chlorobium tepidum*	Thermophile	3H08 [31]	1.60	1.95	49%	69 [31]
ttRNH	*Thermus thermophilus*	Thermophile	1RIL [26]	2.80	1.44	55%	89 [27]
hsRNH	*Homo sapiens*	Mesophile	2QK9 [38]	2.55	2.53	34%	nd^a^

Properties of the RNase H proteins and the crystal structures used to initiate the trajectories described herein. 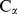 RMSD and percent sequence identity are relative to ecRNH.

a *nd*, not determined.

**Table 2:** Available kinetic measurements for RNase H homologs.

Protein	Temperature ( 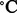 )	Substrate	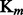 ( 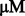 )	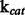 ( 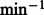 )	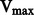 (units/mg)	Reference
soRNH	30	M13 DNA/RNA hybrid	0.30	–	8.6	[55]
ecRNH	30	M13 DNA/RNA hybrid	0.11	–	9.5	[55]
ecRNH	30	Nonanucleotide duplex	0.53	90	–	[25]
ttRNH	30	Nonanucleotide duplex	3.9	19	–	[25]
ecRNH	37	M13 DNA/RNA hybrid	0.11	–	36	[25]
ttRNH	37	M13 DNA/DNA hybrid	0.5	–	7.5	[25]
ttRNH	70	M13 DNA/RNA hybrid	1.1	–	104	[25]

Kinetics data measured under various conditions for soRNH, ecRNH, and ttRNH.

**Figure 1:**
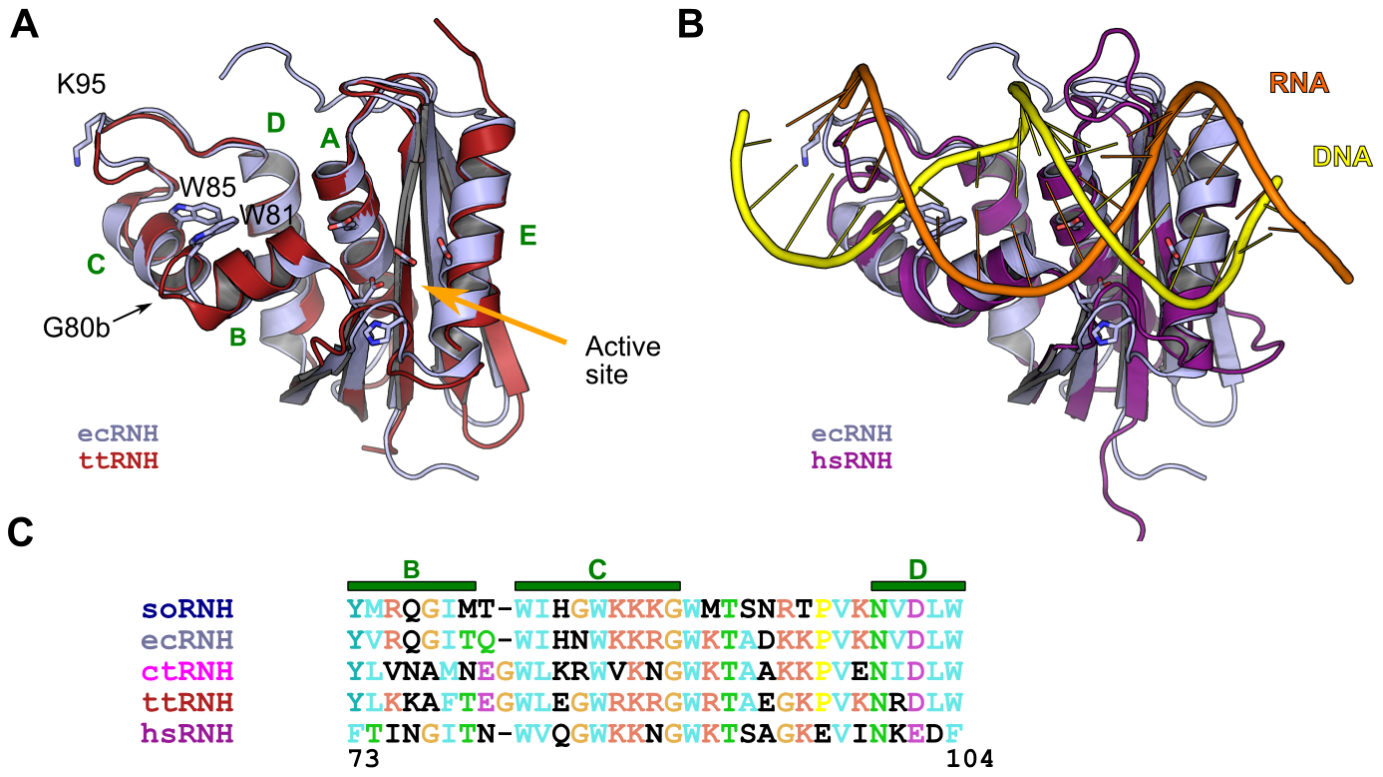
Key features of RNase H structure and sequence. (A) Structural superposition of ecRNH (light blue; PDB ID 2RN2) and ttRNH (red; PDB ID 1RIL). Helices are labeled with green letters and key residues in the handle region and active site (orange arrow) are shown as sticks. (B) Superposition of the ecRNH structure (light blue) with the substrate-bound complex of the hsRNH protein (purple; PDB ID 2QK9), illustrating the position of the handle region interacting with the DNA strand (yellow) of the DNA:RNA hybrid substrate. (C) Sequence alignment of helices B, C, and the handle loop for all five homologs studied.

Key features of the structure of RNase H are illustrated in Figure 1; of particular note is the region of the protein encompassing helices B and C and the following loop, which is known as the handle region or the basic protrusion due to its density of positively charged residues. Although some RNase H homologs lack helix C and the handle loop altogether [33], and ecRNH has been shown to retain some activity when this region is deleted [34], biochemical evidence clearly associates the region with substrate binding [35]–[37]. A naturally handle-less homologous subdomain from the HIV retroviral reverse transcriptase lacks activity in isolation, but an insertion mutant containing the ecRNH handle sequence regains activity under some conditions [35], [36]. Alanine scanning mutations in helix C and the handle loop identify several conserved tryptophan residues critical for binding and reveal that neutralizing positively charged residues in the handle additively disrupts binding affinity [37]. Moreover, crystal structures of the *Homo sapiens* homolog (hsRNH) in complex with substrate show extensive contacts between the DNA strand of the substrate and residues located in helix C and the handle region [38]. Additionally, NMR relaxation measurements suggest that the handle region and a second long loop near the active site have similar rates of motion on the 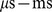 timescale, suggesting a coupled motional process [39].

Two sites near the handle region have been previously identified as major contributors to the differences between ecRNH and ttRNH. First, an inserted glycine, numbered G80b, is present in ttRNH in the junction between helices B and C. NMR studies of ecRNH and ttRNH show increased chemical exchange in the handle region for ttRNH, indicating motion on a 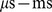 timescale [40]. Reciprocal mutations reveal that the glycine insertion mutant ecRNH iG80b possesses thermophile-like relaxation behavior and significantly impaired catalytic activity; on the other hand, the deletion mutant ttRNH dG80b possesses mesophile-like relaxation behavior, although its activity does not increase [26], [39]. Second, a site at the tip of the handle loop with a conserved left-handed helical conformation in Ramachandran space is occupied by a lysine in ecRNH and a glycine in ttRNH. The ecRNH K95G mutant increases thermostability by 1.9 kcal/mol, likely due to the elimination of the steric strain associated with non-glycine residues in left-handed conformations [41].

Despite this extensive history, the relationships between dynamics, thermostability, and enzymatic activity in the RNase H family remain obscure. In this work we integrate previous NMR observations of handle-region dynamics in ecRNH and ttRNH into an interpretive framework derived from molecular dynamics simulations of all handle-region-containing family members of known structure. These results illustrate the utility of combined MD-NMR studies in elucidating the effects of particular amino acid residues on molecular adaptation to features of the bulk environment.

## Results

**Figure 2:**
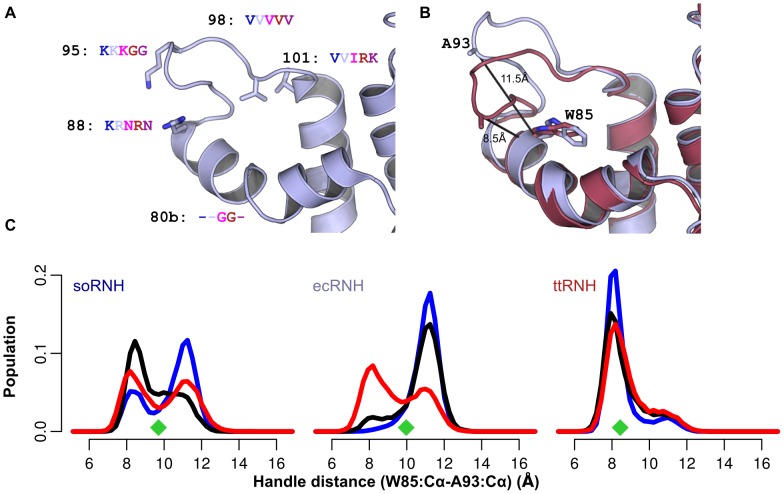
Dynamics of the RNase H handle region as a function of temperature. (A) Key residues modulating handle region dynamics and their identities in each homolog (soRNH, dark blue; ecRNH, light blue; ctRNH, magenta; ttRNH, red; hsRNH, purple). (B) Representative conformations from the ecRNH trajectory of the open (blue) and closed (brown) states, illustrating the Cartesian distance metric used as a reaction coordinate. (C) Temperature dependence of soRNH (left), ecRNH (middle), and ttRNH (right) handle-region dynamics illustrating the relative populations of the closed and open states at 273K (blue), 300K (black), and 340K (red). Measurements of the distance metric from each crystal structure are shown as green diamonds.

Here we present a comparative analysis of molecular dynamics simulations of five homologous proteins with differing thermostabilities and activities at ambient temperature. The simulations provide a structural description of conserved dynamic processes in the RNase H handle region, generating new insight into the role of these motions in substrate binding and identifying key residues responsible for modulating these processes. In addition to the two residues previously known to significantly affect activity and thermostability in RNase H homologs, we find three additional sites in the handle region that are shown by MD to affect local dynamics (Figure 2A). Two sites, V98 and V101 in ecRNH, form part of a hydrophobic cluster that also includes the two conserved Trp residues, W81 and W85, known to directly interact with substrate. The third, R88 in ecRNH, is conserved in its best-studied homolog ttRNH, but here is shown to be a critical determinant of handle region dynamics among the larger RNase H family.

### Two-state behavior in the handle region

We begin with the three proteins containing an arginine or lysine residue at position 88 at the end of helix C: soRNH, ecRNH, and ttRNH. The motion of the handle region in each protein is monitored by a reaction coordinate consisting of a simple Cartesian distance metric between the 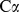 atoms of A93 at the tip of the handle loop and W85 as an anchor point on helix C (ecRNH residues and numbering), as illustrated in Figure 2B and Figure S1 and plotted as a function of simulation time for representative trajectories in Figure S2. These three proteins share a conserved dynamic mode in which two distinct handle conformations are observed, an open and closed state. The open state is populated by soRNH and ecRNH at lower temperatures, while elevated temperatures simply equalize the populations of each state, as expected. In contrast, ttRNH predominantly occupies the closed conformation at all temperatures studied. Notably, the corresponding distances in the crystal structures of all three proteins lie between the two conformations observed in the simulations (Figure 2C), possibly due to the presence of crystal contacts in that region (Figure S3A) or an inability to model both states during crystallographic refinement. The thermostability of the ecRNH protein has been extensively studied by mutagenesis; a survey of these ecRNH mutant structures, though dominated by contact-stabilized intermediate conformations, also identifies examples of both the open and closed conformations (Figure S3B). Preference for the open conformation among the two more active homologs suggests that this may be the conformation competent for substrate binding. We hypothesize that ttRNH is reliant on thermal fluctuations to access the open conformation on a timescale exceeding that studied here. This pattern is reminiscent of observations previously made in triose phosphate isomerase [6], dihydrofolate reductase [42], and adenylate kinase [43], in which simulations suggest rapid, nanosecond-timescale sampling of partially activated conformations, but a stable fully activated conformation is suggested by experiment to be accessible only at millisecond timescales.

The ecRNH and ttRNH simulations can be validated by comparison to experimental NMR data. Calculated 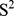 order parameters, reflecting amplitude of local motion, are in good agreement with the experimental values for both proteins (Figure S4). In addition, we have previously shown that simulation-derived chemical shift predictions reflecting dynamic conformational averaging perform significantly better than predictions from the static crystal structures in reproducing experimental chemical shift data for ecRNH and ttRNH [44]. This agreement is particularly significant because chemical shifts, especially those of protons, are highly sensitive to ring-current effects from the orientation of aromatic groups, which are plentiful near the handle loop. The accuracy of dynamically averaged predictions of chemical shifts for these two proteins (Figure S5) supports the hypothesis that the motions observed in the 300K simulations recapitulate motions observed experimentally. The handle loop typically shows below-average RMSDs to the experimental chemical shift values (Table 3), suggesting that this particularly dynamic region is reasonably well-sampled.

**Table 3:** Chemical shift RMSDs for flexible regions of ecRNH and ttRNH.

	ecRNH				ttRNH			
Protein region	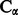	HN	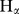	N	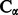	HN	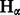	N
Handle (MD)	0.45	0.42	0.31	2.34	0.38	0.42	0.25	1.97
Handle (Xray)	0.38	0.47	0.27	2.51	1.16	0.51	0.28	4.02
Average (MD)	0.70	0.36	0.25	2.25	0.68	0.35	0.23	2.17
Average (Xray)	0.74	0.39	0.25	2.51	1.00	0.44	0.32	2.70

RMSDs for the 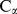 and N Sparta+ chemical shift predictions to experimental values for ecRNH [57] and ttRNH [40]. The improvement due to dynamic averaging is particularly good in the handle region for the relatively low-resolution ttRNH structure, while the ecRNH values are within the magnitude of the error of the predictor.

**Figure 3:**
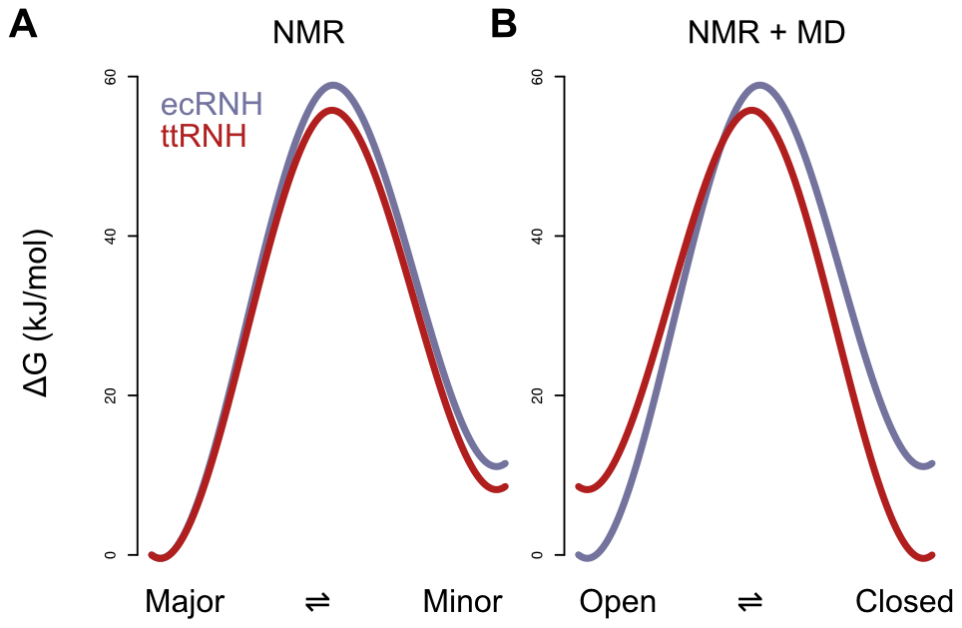
Free-energy landscapes for putative major and minor states for ecRNH and ttRNH. (A) The free-energy diagram constructed under the assumption that both ecRNH and ttRNH share a major state that is incompetent for substrate binding [39]. (B) A revised diagram, inspired by the simulated populations, in which the landscape for ttRNH is mirrored, suggesting that the (closed) minor state of ecRNH is equivalent to the major state of ttRNH, and the (open) major state of ecRNH is equivalent to the minor state of ttRNH.

Previous NMR relaxation measurements on ecRNH and ttRNH produced estimates of the relative free energies of major and minor conformational states, summarized in the free-energy diagram in Figure 3A [39]. This landscape was constructed based on the observations that a) the ecRNH and ttRNH crystal structures closely resemble one another, and b) those structures do not appear to be in a binding-competent conformation. However, this result was perplexing because the putatively binding-competent state was more highly populated in the less-active ttRNH. Population estimates from the simulations suggest an alternative interpretation (Figure 3): that the minor state of ecRNH at 300K is equivalent to the major state of ttRNH, and vice versa; thus, a mirrored version of our original free energy profile is likely a better representation of the experimental data. While it is unlikely that such short simulations reproduce equilibrium behavior, the overall picture of a conserved dynamic process with a larger activation barrier in ttRNH is consistent with previous observations [40].

### An alternative mode of substrate binding

**Figure 4:**
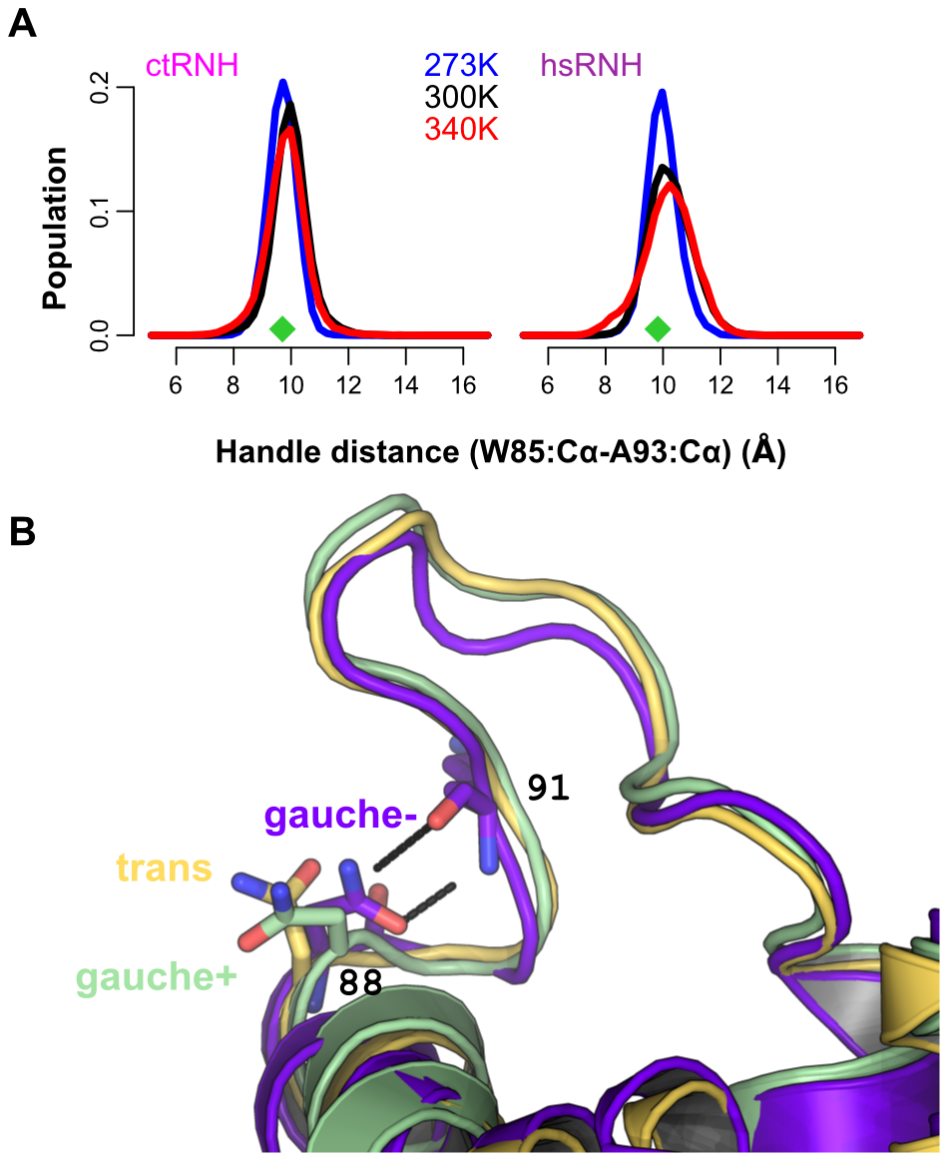
Dynamics of the handle region in the presence of Asn at position 88. (A) Temperature-dependent populations for the two natively Asn-containing proteins—ctRNH (left) and hsRNH (right) —illustrating the presence of a single conformationaln distribution centered around the crystal-structure position (green diamonds) whose basin broadens with increasing temperature. (B) The presence of Asn at position 88 permits the formation of two highly stable hydrogen bonds to the backbone of residue 91 when Asn occupies the *gauche-* 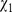 rotamer; the ecRNH R88N mutant, whose trajectory samples all three states, is shown here.

Two proteins in our data set, ctRNH and hsRNH, contain an asparagine at residue 88, where the other proteins contain arginine or lysine. The natively Asn-containing proteins do not exhibit two-state behavior, but instead show a single peak for the handle-region metric, centered around the crystal structure value and broadening with increasing temperature (Figure 4A). Although hsRNH was crystallized in complex with substrate and might be thought to occupy a distinct handle-region conformation due to substrate interactions, the average all-to-all 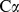 RMSD between the handle regions in the 300K trajectories of hsRNH and ctRNH, which was crystallized without substrate, is only 1.04 Å.

To explore the effects of asparagine and arginine on handle-region behavior, we made mutations at this site for all five proteins. In four cases, the resulting mutants are stable under the simulation conditions at 300K for 100 ns, but hsRNH N88R requires two additional stabilizing mutations: in the prokaryotic proteins, a pair of well-conserved residues, Y73 and W104, anchor the interface between helices B and D; in hsRNH, both are replaced by Phe. The absence of the additional hydrogen bonding contributions in hsRNH N88R disrupts the interfaces between helices B, D, and A (Figure S7A–B); however, the triple mutant hsRNH F73Y/N88R/F104W is stable and shows dynamics similar to those observed for the prokaryotic homologs.

**Figure 5:**
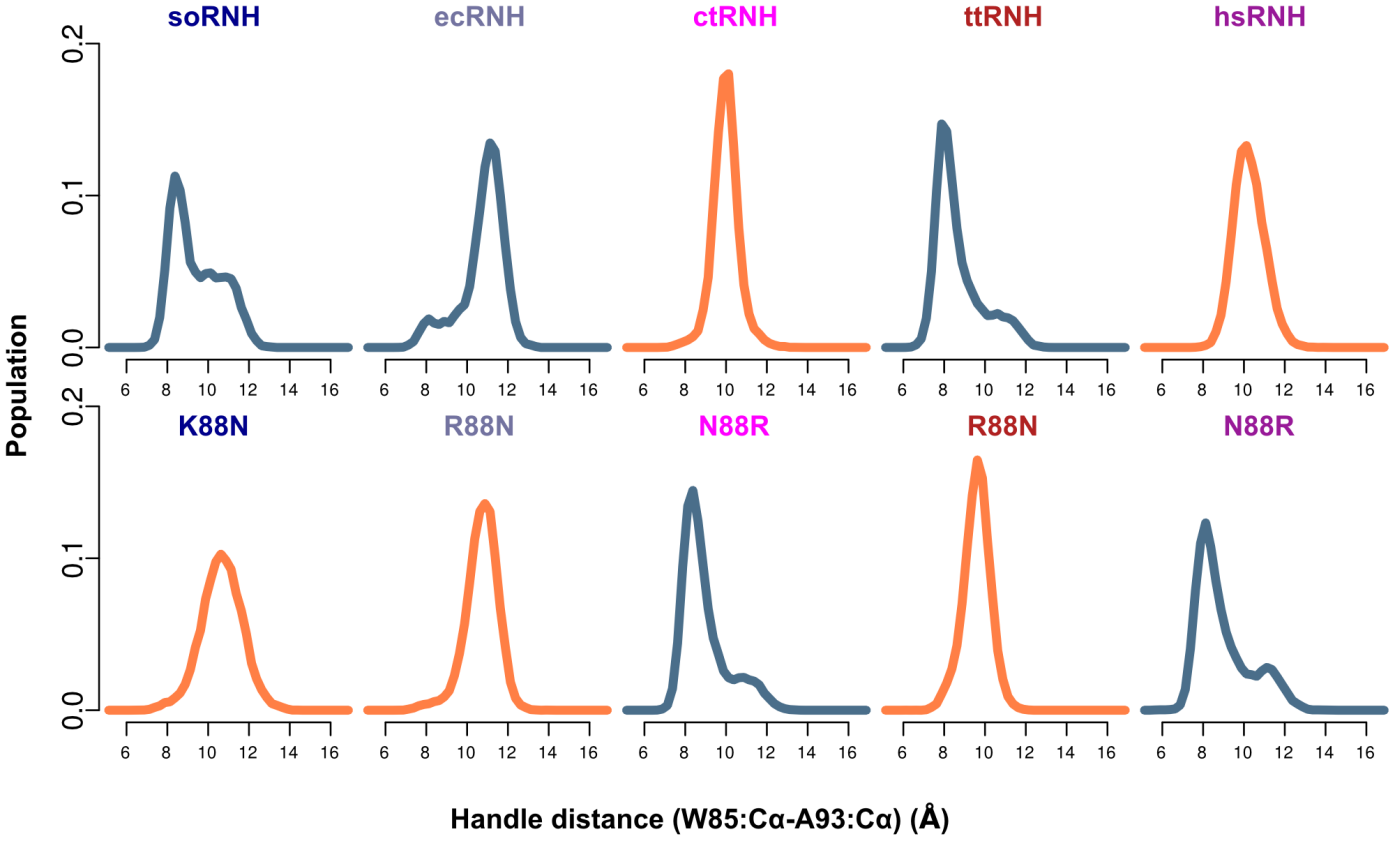
Handle-region behavior for reciprocal mutants at position 88. The top row shows wild-type proteins and the bottom shows each protein's corresponding mutant. Distributions shown in blue indicate the presence of a positively charged residue at position 88 (Arg in all cases except soRNH, which natively contains Lys) and distributions shown in orange indicate the presence of Asn at this position. All simulations were carried out at 300K.

The dynamic consequences of substitutions at position 88 are clearly shown in Figure 5: when Arg or Lys occupies this site, the handle region shows two-state behavior, while Asn produces a single handle-distance peak centered roughly between the open and closed states for the two-state systems. In both wild-type and mutant proteins containing Asn, a dominant *gauche-* 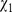 rotameric state for this residue is observed in which the sidechain amide forms two hydrogen bonds to the backbone carbonyl and amide of the neighboring residue at position 91 (Figure 4B). By contrast, Arg 88 is highly flexible and forms only transient, often water-mediated hydrogen bonds with its neighbors; the sidechain order parameter for Arg 88 in ecRNH has been measured as around 0.2 at 300K, and this low value is well-reproduced in simulation [45].

### Tuning handle region populations

The remaining four residue positions highlighted in Figure 2A are identified by the simulations as critically important in determining the relative populations of the open and closed states. Three of these sites—the glycine insertion (G80b), Val 98, and Val 101 (ecRNH residues and numbering)—form the borders of a hydrophobic spine linking helices C and D through two conserved Trp residues involved in direct substrate contacts. In ecRNH, rotamer jumps at the two valine sites correlate with both predicted chemical shift and the handle-distance metric (Figure S6). The remaining site, Lys 95, resides at the tip of the handle loop and requires a left-handed helical backbone conformation. Strategic substitutions at these sites allow us to rationally manipulate the relative populations of the open and closed states in both native and mutant proteins with a positively charged residue at position 88.

**Figure 6:**
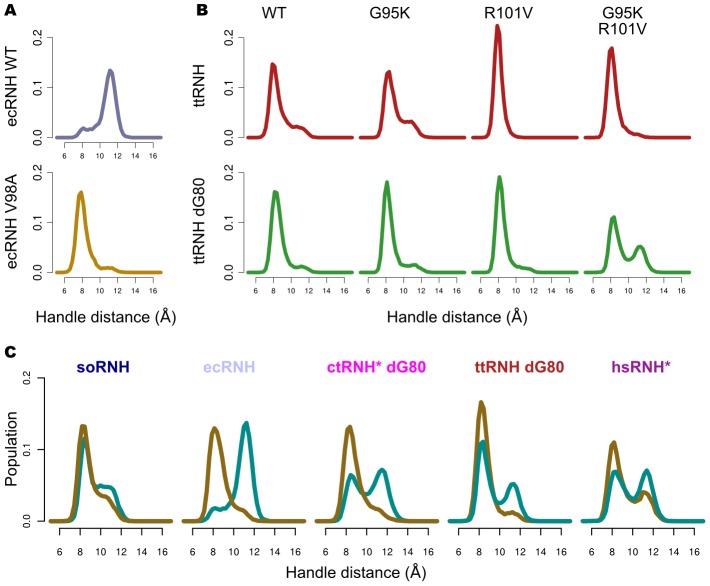
Coupling of handle-region dynamics to residues in the handle region. (A) Handle-region distance distributions for ecRNH WT (top) and V98A (bottom), illustrating the predominance of the closed state in the mutant. (B) Coupled effects of mutations at positions 95 and 101 in ttRNH (top) and ttRNH dG80 (bottom). Only ttRNH dG80 G95K/R101V shows a population of the open state significantly enriched compared to wild-type ttRNH. (C) Manipulation of relative populations by coupled mutations at positions 95 and 101. For all arginine- or lysine-containing proteins other than soRNH, mutants containing G95 and R101 (brown) populate the closed state more frequently than those containing K95 and V101 (cyan), regardless of the wild-type residues at these positions. The natively N88-containing proteins, ctRNH and hsRNH, both required additional mutations to stabilize the interface between helices B and D, as detailed in Figure S7.

Position 98 is highly conserved as a Val among prokaryotic RNases H that possess handle regions, underscoring its functional significance, despite the lack of direct contact between its sidechain and substrate. The mutant ecRNH V98A abrogates the observed rotamer transitions and populates a predominantly closed conformation (Figure 6A).

In ecRNH, rotameric transitions of Val 101 induce subtle changes in local packing throughout the hydrophobic spine, potentially stabilizing the open conformation. To produce a ttRNH mutant with increased population of the open state, we therefore made reciprocal mutations at this position in both the presence and absence of the inserted Gly at position 81 and the left-handed Gly residue at position 95, which is occupied by a Lys in ecRNH. The results of these mutations are summarized in Figure 6B. In brief, the mutations work in concert; while no single mutant significantly increases open state population, a ttRNH dG80/G95K/R101V triple mutant populates the open state at a level of about 40%, compared to about 10–15% for the wild-type and dG80 enzymes. Conversely, an ecRNH K95G/V101R/Q105E mutant enriches population of the closed state relative to wild type. (In this case a double mutant was necessary to provide the Arg with an equivalent to its native hydrogen-bonding partner.) The success of these mutations in altering the local conformational equilibrium underscores the importance of this hydrophobic cluster.

Notably, corresponding mutations in the context of the hsRNH F73Y/N88R/F104W triple mutant produce the same effects on its open-closed dynamics. The wild-type hsRNH protein lacks a glycine insertion but contains a Gly at position 95 and a Lys at position 101, similar to the ttRNH protein. The quintuple mutant obtained by the additional G95K/K101V substitutions significantly increases the population of the open state relative to the triple mutant. Similarly, ctRNH dG80/N88R is predicted from its sequence—K95, I101—to predominantly populate the open state in solution. This protein, like hsRNH, required reengineering of the interface between helices B and D to form a stable structure (Figure S7C–D); the modified form of the protein behaves as predicted, populating the open conformation more frequently with the native K95/I101 residues than with the mutant G95/R101 (Figure 6C).

## Discussion

We identify two conserved dynamic modes in the handle region of RNase H, determined by the identity of a single residue at position 88 at the C-terminus of helix C: when this site is Arg or Lys, a two-state equilibrium between open and closed states is observed, while an Asn at this site stabilizes a single state roughly intermediate between the extremes defined by the open and closed states. The handle loop has previously been suggested to move as a rigid body in ecRNH and ttRNH; these results suggest that it can either swing on loose hinges, or be buttressed by the sidechain-backbone hydrogen bonds for which an Asn residue at this site is uniquely well-suited. A suppressor screen for thermostabilizing mutations of soRNH, which natively contains Lys at this position, identified K90N as thermostabilizing by 0.7 kcal/mol with only a 9% decrease in activity relative to the wild-type protein [29], consistent with our observations by computational mutagenesis that these reciprocal mutations are mostly nondisruptive and are easily accommodated in the local environment. Interestingly, among bacterial proteins containing handle loops, the frequency of ocurrence of Asn is higher among those sequences annotated as having a thermophilic source organism than among those annotated as being derived from mesophiles (Figure S9).

Among the two-state proteins—soRNH, ecRNH, and ttRNH—a trend is observed favoring population of the open state at temperatures near those preferred by the source organism. In particular, the thermophile-derived ttRNH does not significantly populate the open state at ambient temperatures and likely relies on thermal fluctuations to surmount the energy barrier between states. Although neither conformation is well-positioned geometrically to receive substrate by comparison to the hsRNH complex structure, this pattern suggests that the open state is likely to be the binding-competent state. Relatively subtle changes in local hydrophobic packing accompany open-to-closed transitions in a loosely coupled manner and can be exploited by mutagenesis to tune the relative populations. Two conserved tryptophan residues, W81 and W85, in the hydrophobic cluster manipulated by these mutations are known to form close contacts with substrate and likely require precise positioning for productive interactions. Notably, sites previously identified as relevant to thermostabilization among RNase H proteins—positions 80b and 95—play an important role in cooperatively determining relative populations of open and closed states. For sites 80b, 95, and 101, weak trends are observed among available RNase H sequences favoring the residues that contribute to increased closed-state population among sequences annotated as derived from thermophilic organisms (Figures S8, S9), suggesting that adaptation to high-temperature environments directly trades off against population of the open state. These results suggest that mesophilic organisms tolerate thermally destabilizing non-glycine residues in the left-handed dihedral conformation structurally required at position 95 due to their effects on relative open-state population.

**Figure 7:**
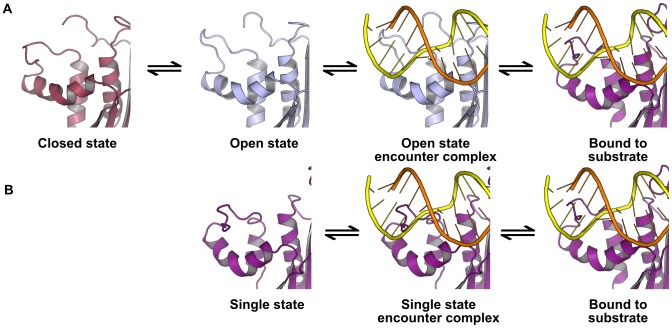
Kinetic schemes for substrate binding in one- and two-state proteins. (A) The kinetic scheme for the interaction of substrate with a two-state handle region, where the open state is the binding-competent state. (B) The kinetic scheme for a single-state handle region, in which the loop is held in a single conformation well-positioned for substrate interactions.

Several studies have demonstrated the close relationship between dynamics observed in an enzyme's apo state and those observed in substrate complexes [46], [47] . Differences in the conformational dynamics of the apo states of homologous proteins could therefore contribute to differences in the kinetics of substrate binding or product release. The binding kinetics of the two classes of RNase H homologs identified here, differentiated by the residue at position 88, are predicted to differ significantly. The kinetic scheme for two-state proteins is a two-step process: a conformational selection step in which the substrate binds preferentially to the open state is followed by an induced fit process in which the open handle loop rearranges to form hydrogen-bonding interactions with the DNA strand of the substrate (Figure 7A). Because the RNase H protein must discriminate not only between different types of nucleic acids, but also between the two strands of its hybrid substrate, a two-step process in which an encounter complex quickly dissociates if the strands are misaligned could provide significant regulatory advantage. Altering the relative population of the open state through mutation at sites not directly involved in the substrate-binding interface offers a means for fine-tuning conformational preferences to match both the functional context and the thermal environment. By contrast, the kinetic scheme for the single-state, Asn-containing proteins is a single-step process, as the loop conformation stabilized by Asn-backbone hydrogen bonds is already oriented for productive interactions with substrate (Figure 7B).

Collectively, these results suggest that, despite high sequence homology among the RNase H proteins studied here, the protein fold permits multiple possible adaptive pathways to balance the competing constraints represented by conformational dynamics and thermostabilization.

## Materials and Methods

Simulations were performed using Desmond Academic release 3 or source release 2.4.2.1 [48]. Proteins were described with the Amber99SB force field [49] , solvated with TIP3P water in a cubic box with a 10 Å buffer region from solute to box boundary, and neutralized with 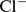 ions. Electrostatics were calculated with the PME method. All simulations used a 2.5fs inner timestep on a 1-1-3 RESPA cycle and were performed in the NVT ensemble using a Nosé-Hoover thermostat after equilibration to constant box volume in the NPT ensemble.

The PDB structures 2E4L, 2RN2, 3H08, 1RIL, and 2QK9 were used to initiate trajectories at each of 273K, 300K, and 340K for wild-type simulations run for 100 ns each. Computational mutagenesis on these structures was performed in Maestro version 9.1 for solvent-exposed sites or MODELLER v9.5 for packed sites. All structures were protonated in accordance with H++ [50] pKa predictions to replicate the pH of 5.5 used in previous NMR experiments on ecRNH and ttRNH. Crystallographic waters were removed and all structures solvated using Maestro version 8.5 or 9.1. For 2QK9, the substrate was removed and the catalytically inactivating D210N mutation reversed in Maestro. For 3H08, missing residues were modeled in using MODELLER with 1RIL as a template; 273K and 340K trajectories for this protein were initiated from a randomly selected frame from its 300K trajectory. For the ttRNH dG80 mutant, a crystal structure was not available; trajectories were initiated from a model produced in MODELLER using 1RIL and 2RN2 as templates. Chemical shift predictions were performed as described [44]; RMSD to experimental values for the dG80 trajectory is of similar magnitude as that for the trajectories initiated from crystal structures. Handle-region dynamics were monitored using a reaction coordinate consisting of the Cartesian distance between the residues equivalent to W85 and A93 in ecRNH; values greater than 10 Å were considered to reflect an open state. Order parameters were calculated using the equation 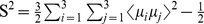 [51]. Images were prepared in PyMol.

Sequences of bacterial RNase H domains were collected from InterPro entry IPR002156 [52] and annotated for source organism growth temperature using the Integrated Microbial Genomes database [53]. Sequences that were redundant or did not contain a handle loop were removed and the remaining sequences aligned to the four available bacterial structures using PROMALS3D [54].

## Supporting Information

Figure S1
**Principal components analysis of the handle loop for all five RNase H proteins.** PCA analysis on the 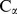 Cartesian coordinates of the handle loop, corresponding to residues G89 to N100 in ecRNH, was carried out on the 300K trajectories of all five wild-type proteins. Projections onto the first two principal components are shown for soRNH (dark blue), ecRNH (light blue), ctRNH (magenta), ttRNH (red), and hsRNH (purple); crystal structures are indicated as filled circles. The first principal component axis describes the difference between single-state and two-state proteins, while the second describes the difference between the open and closed states. Collectively these two principal components account for 89% of the variance in the dataset.(TIFF)

Figure S2 **Timecourses of handle region dynamics for ecRNH and ttRNH.** The fluctuations of the handle-region distance metric as a function of time are shown for ecRNH (left; blue) and ttRNH (right; red) for the 300K trajectories, representing 100 ns of simulation time.
(TIFF)

Figure S3 **Crystal contacts identified in ecRNH structures.** (A) The crystal-packing environment surrounding the ecRNH handle region (blue) in 2RN2. Symmetry mates are shown in green, yellow, and brown; the local hydrogen bonding network is shown as black lines. (B) Distribution of handle-distance measurements in 54 chains representing 32 PDB structures of the ecRNH protein.
(TIFF)

Figure S4
**Predicted vs. experimental backbone amide order parameters.** (A) Comparison between experimental [58] (black) and predicted (blue) 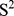 order parameters for ecRNH. Helices B, C, and the handle region are highlighted in green. (B) Comparison between experimental [40] (black) and predicted (red) order parameters for ttRNH. Correlations as determined by Pearson's R are 0.89 and 0.74 respectively; the lower correlation for ttRNH is likely due to the fact that the experimental values were acquired at 310K using a cysteine-free form of the protein to avoid undesirable thiol chemistry. Experimental values have been rescaled by the slope of a linear regression to the simulated values for visualization.(TIFF)

Figure S5
**Dynamically averaged chemical shift predictions.** (A) Comparison between experimental (black) and predicted (blue) secondary chemical shift values for the nuclei with the smallest (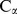) and largest (N) RMSD values among those predicted in [44] for ecRNH. Helices B, C, and the handle region are highlighted in green. (B) Comparison between experimental (black) and predicted (red) secondary chemical shift values for ttRNH. Predicted values are reproduced from [44]. Values are plotted as secondary chemical shifts (deviation from random-coil value for each residue); RMSDs are calculated using the absolute shift values.(TIFF)

Figure S6
**Coupling of handle-region dynamics to valine rotamers in ecRNH.** (A) Correlation between the handle distance metric and the 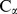 chemical shift of V98 in ecRNH. Structures at right indicate the most common V98 rotamer giving rise to the corresponding chemical shift value. Points are colored from dark to light blue to reflect the timecourse of the trajectory. (B) Correlation between the handle distance and the N chemical shift of V101 in ecRNH.(TIFF)

Figure S7 **Mutations introduced to stabilize the helix B-helix D interface in N88R mutants.** (A) Superposition of ecRNH (blue) and hsRNH (purple), illustrating the phenylalanines mutated in hsRNH* to their homologous bacterial residues. (B) Destabilized conformation of hsRNH N88R in the absence of the hsRNH* mutations F73Y/F104W. (C) Model of ctRNH dG80 mutant ctRNH*, illustrating the mutations made to the packing interface in helix B to construct a stable context into which to make the N88R mutation. (D) Sequence of ctRNH* dG80 helix B.
(TIFF)

Figure S8 **Residue frequencies in the glycine-insertion position.** Distribution of residues among the 198 bacterial RNase H domain sequences identified as possessing an insertion (left); frequency of insertion as a function of growth temperature annotation of the source organism (right).
(TIFF)

Figure S9
**Residue frequencies in sites identified as significant determinants of handle-region dynamics.** Distribution of residues at each of positions 88, 95, and 101 among bacterial RNase H sequences from all organisms, and as a function of growth temperature annotation. For position 101, residues have been clustered into four categories: alanines, branched amino acids (isoleucine, leucine, valine), linear and polar amino acids (arginine, lysine, glutamate, glutamine), and other amino acids. For positions 88 and 101, the notation * indicates a distribution significantly different from uniform, and the notation # indicates a distribution significantly different from the overall dataset (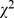 test with Bonferroni-corrected significance level of p<0.003). Mean percent sequence identities for each category are 54% (overall), 62% (psychrophiles), 55% (mesophiles), 51% (thermophiles).(TIFF)
