# Supplementary figures and images for: Thermal Adaptation of Conformational Dynamics in Ribonuclease H
Source: PLoS Comput Biol. 2013 Oct 3;9(10):e1003218. doi: 10.1371/journal.pcbi.1003218 (PMC3789780; doi:10.1371/journal.pcbi.1003218)

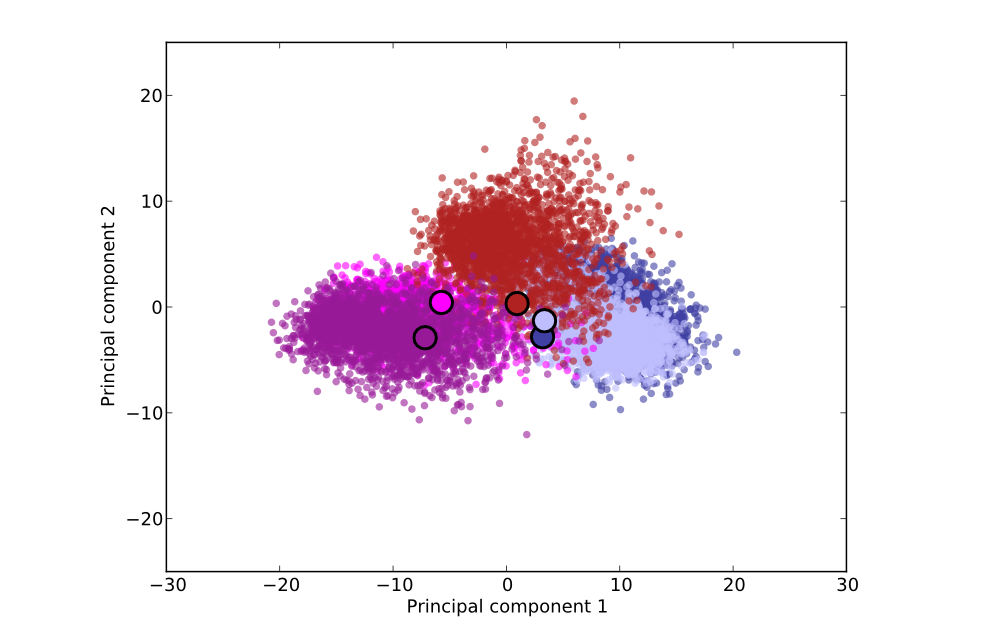

Supplement: Figure S1 — Principal components analysis of the handle loop for all five RNase H proteins. PCA analysis on the Cartesian coordinates of the handle loop, corresponding to residues G89 to N100 in ecRNH, was carried out on the 300K trajectories of all five wild-type proteins. Projections onto the first two principal components are shown for soRNH (dark blue), ecRNH (light blue), ctRNH (magenta), ttRNH (red), and hsRNH (purple); crystal structures are indicated as filled circles. The first principal component axis describes the difference between single-state and two-state proteins, while the second describes the difference between the open and closed states. Collectively these two principal components account for 89% of the variance in the dataset. (TIFF) [file pcbi.1003218.s001.tiff]

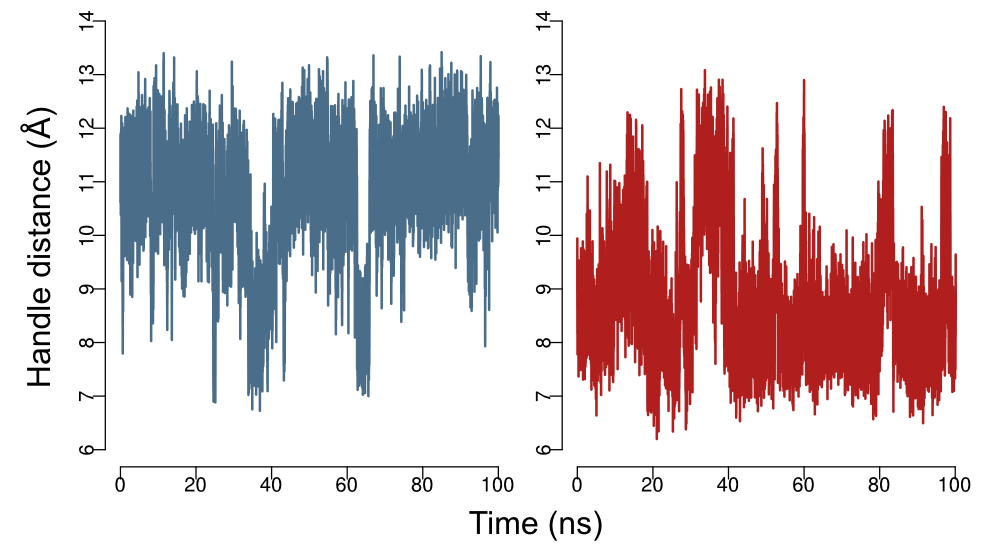

Supplement: Figure S2 — Timecourses of handle region dynamics for ecRNH and ttRNH. The fluctuations of the handle-region distance metric as a function of time are shown for ecRNH (left; blue) and ttRNH (right; red) for the 300K trajectories, representing 100 ns of simulation time. (TIFF) [file pcbi.1003218.s002.tiff]

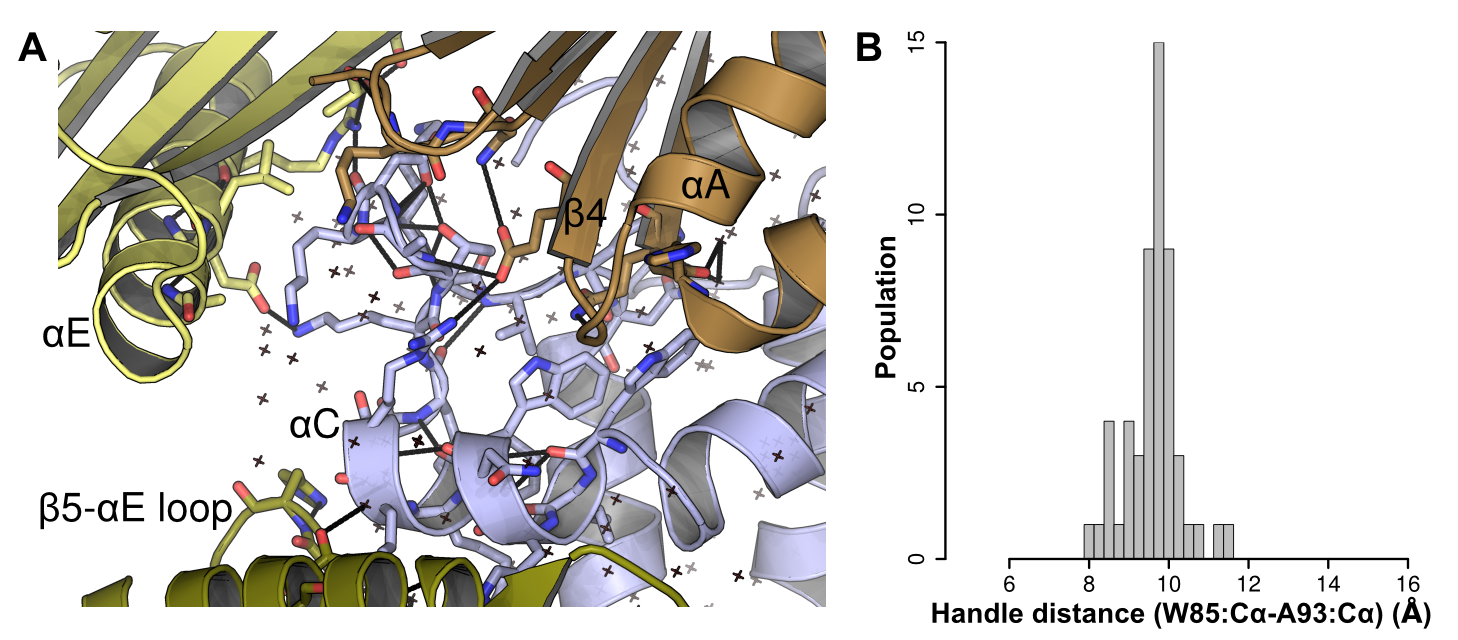

Supplement: Figure S3 — Crystal contacts identified in ecRNH structures. (A) The crystal-packing environment surrounding the ecRNH handle region (blue) in 2RN2. Symmetry mates are shown in green, yellow, and brown; the local hydrogen bonding network is shown as black lines. (B) Distribution of handle-distance measurements in 54 chains representing 32 PDB structures of the ecRNH protein. (TIFF) [file pcbi.1003218.s003.tiff]

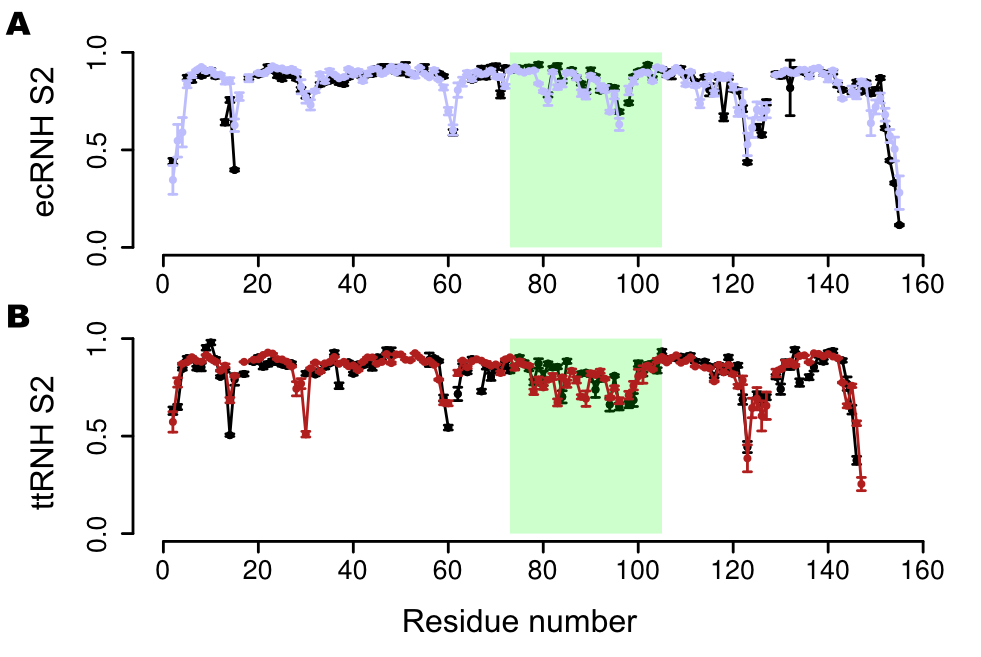

Supplement: Figure S4 — Predicted vs. experimental backbone amide order parameters. (A) Comparison between experimental [58] (black) and predicted (blue) order parameters for ecRNH. Helices B, C, and the handle region are highlighted in green. (B) Comparison between experimental [40] (black) and predicted (red) order parameters for ttRNH. Correlations as determined by Pearson's R are 0.89 and 0.74 respectively; the lower correlation for ttRNH is likely due to the fact that the experimental values were acquired at 310K using a cysteine-free form of the protein to avoid undesirable thiol chemistry. Experimental values have been rescaled by the slope of a linear regression to the simulated values for visualization. (TIFF) [file pcbi.1003218.s004.tiff]

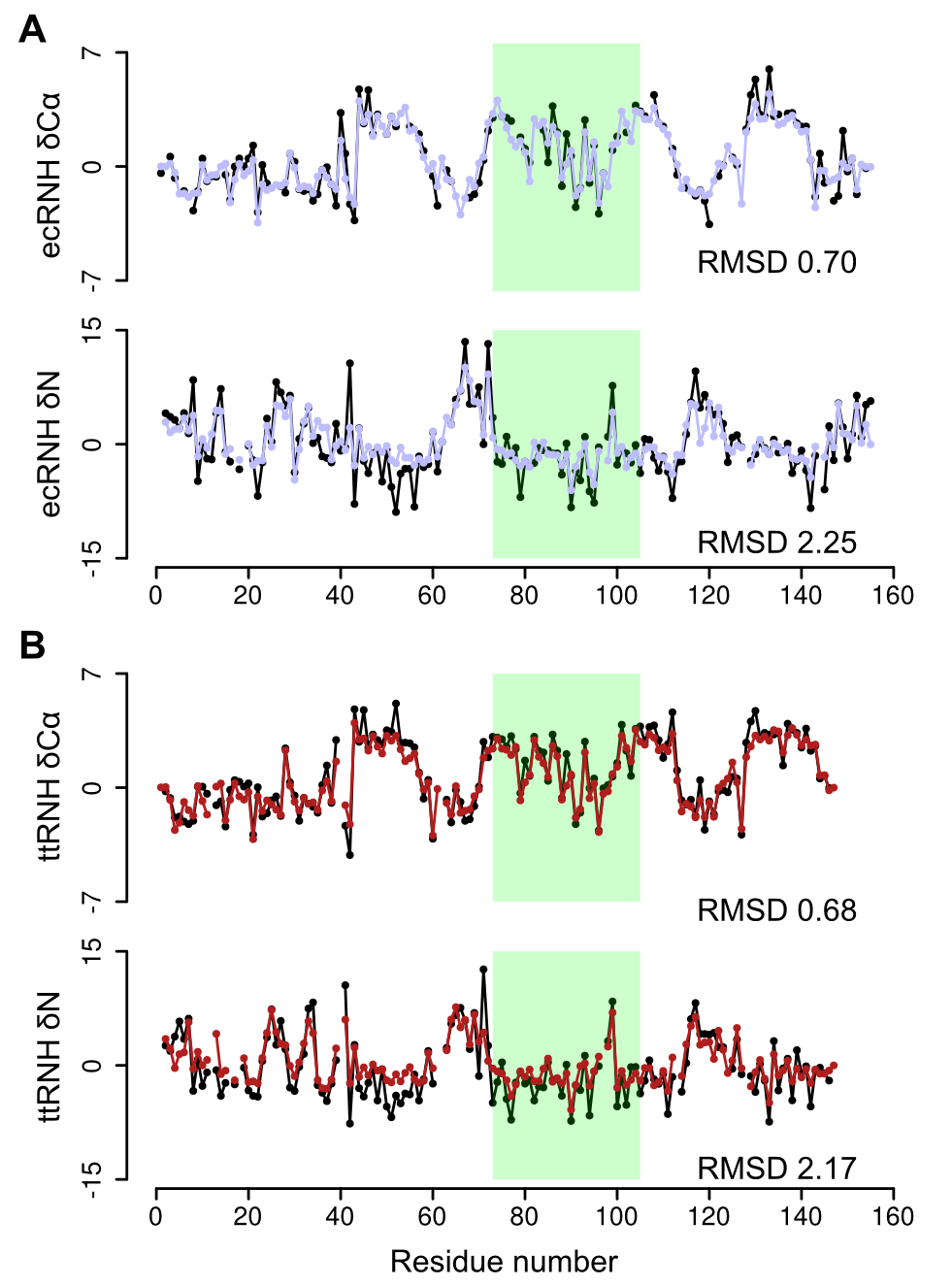

Supplement: Figure S5 — Dynamically averaged chemical shift predictions. (A) Comparison between experimental (black) and predicted (blue) secondary chemical shift values for the nuclei with the smallest () and largest (N) RMSD values among those predicted in [44] for ecRNH. Helices B, C, and the handle region are highlighted in green. (B) Comparison between experimental (black) and predicted (red) secondary chemical shift values for ttRNH. Predicted values are reproduced from [44]. Values are plotted as secondary chemical shifts (deviation from random-coil value for each residue); RMSDs are calculated using the absolute shift values. (TIFF) [file pcbi.1003218.s005.tiff]

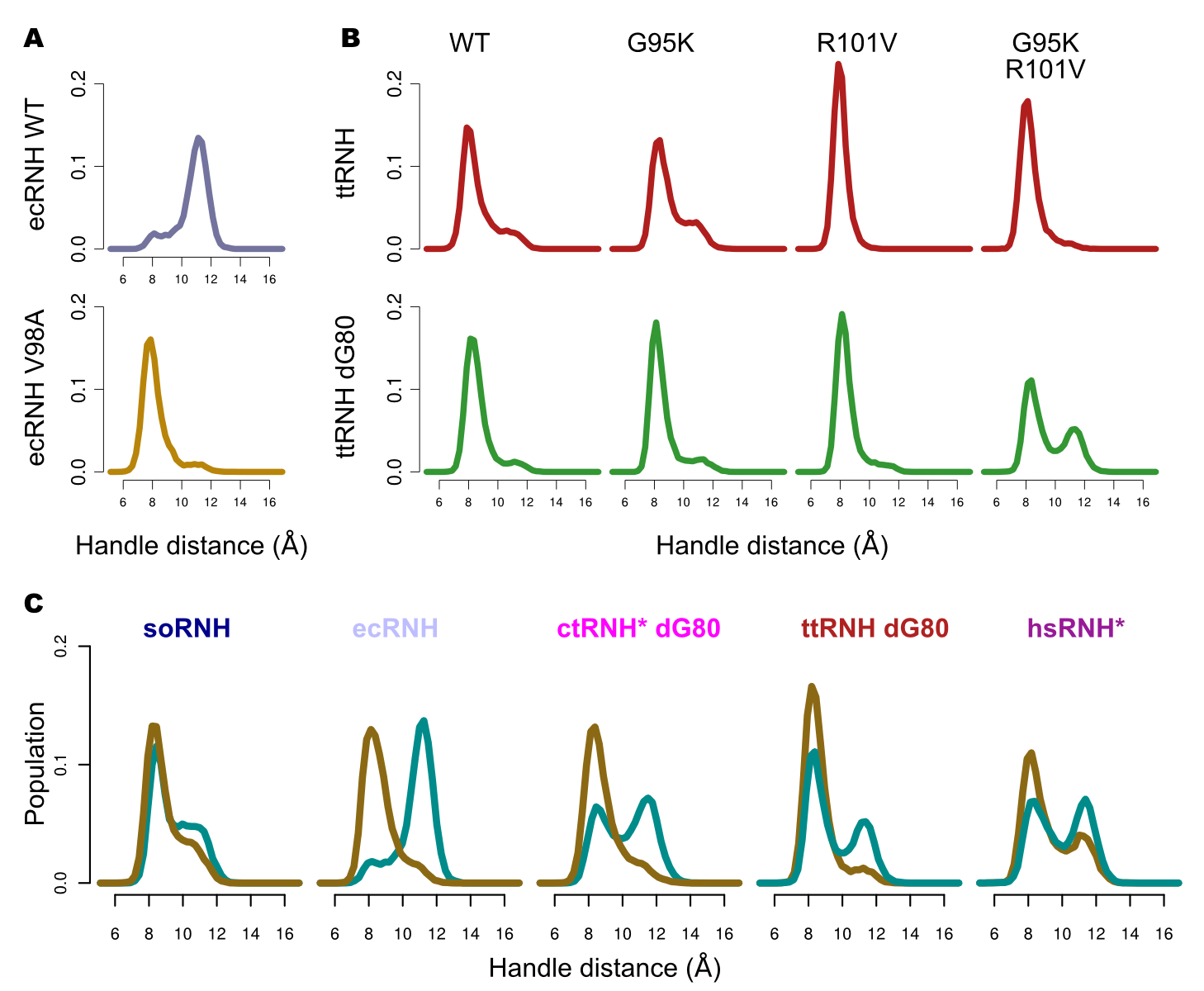

Supplement: Figure S6 — Coupling of handle-region dynamics to valine rotamers in ecRNH. (A) Correlation between the handle distance metric and the chemical shift of V98 in ecRNH. Structures at right indicate the most common V98 rotamer giving rise to the corresponding chemical shift value. Points are colored from dark to light blue to reflect the timecourse of the trajectory. (B) Correlation between the handle distance and the N chemical shift of V101 in ecRNH. (TIFF) [file pcbi.1003218.s006.tiff]

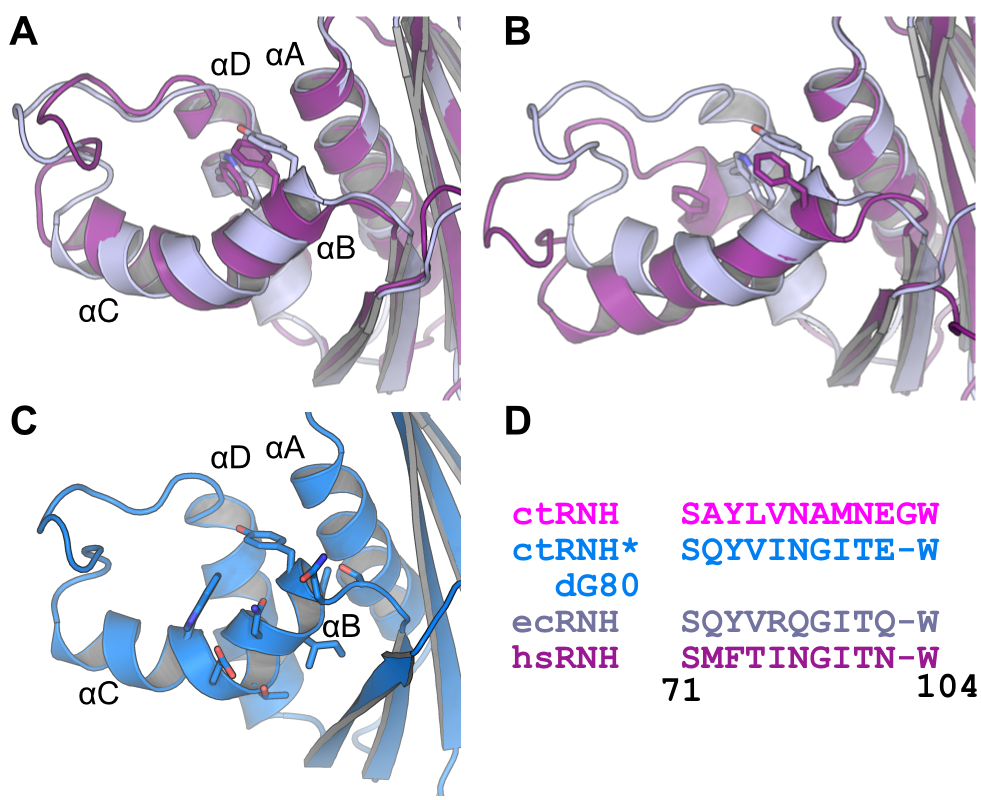

Supplement: Figure S7 — Mutations introduced to stabilize the helix B-helix D interface in N88R mutants. (A) Superposition of ecRNH (blue) and hsRNH (purple), illustrating the phenylalanines mutated in hsRNH* to their homologous bacterial residues. (B) Destabilized conformation of hsRNH N88R in the absence of the hsRNH* mutations F73Y/F104W. (C) Model of ctRNH dG80 mutant ctRNH*, illustrating the mutations made to the packing interface in helix B to construct a stable context into which to make the N88R mutation. (D) Sequence of ctRNH* dG80 helix B. (TIFF) [file pcbi.1003218.s007.tiff]

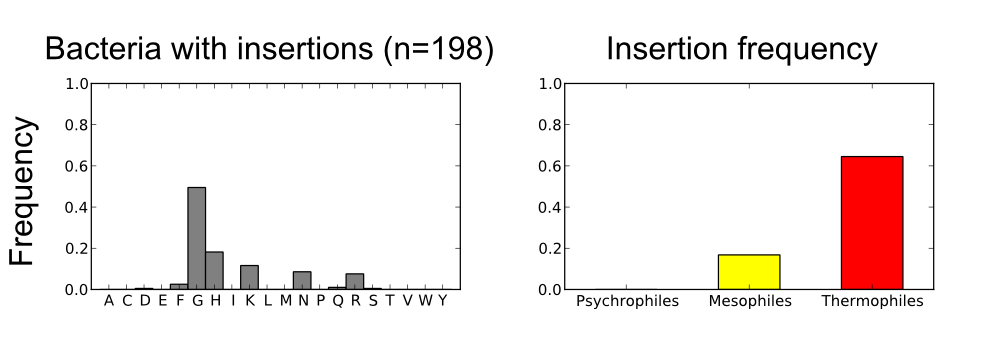

Supplement: Figure S8 — Residue frequencies in the glycine-insertion position. Distribution of residues among the 198 bacterial RNase H domain sequences identified as possessing an insertion (left); frequency of insertion as a function of growth temperature annotation of the source organism (right). (TIFF) [file pcbi.1003218.s008.tiff]

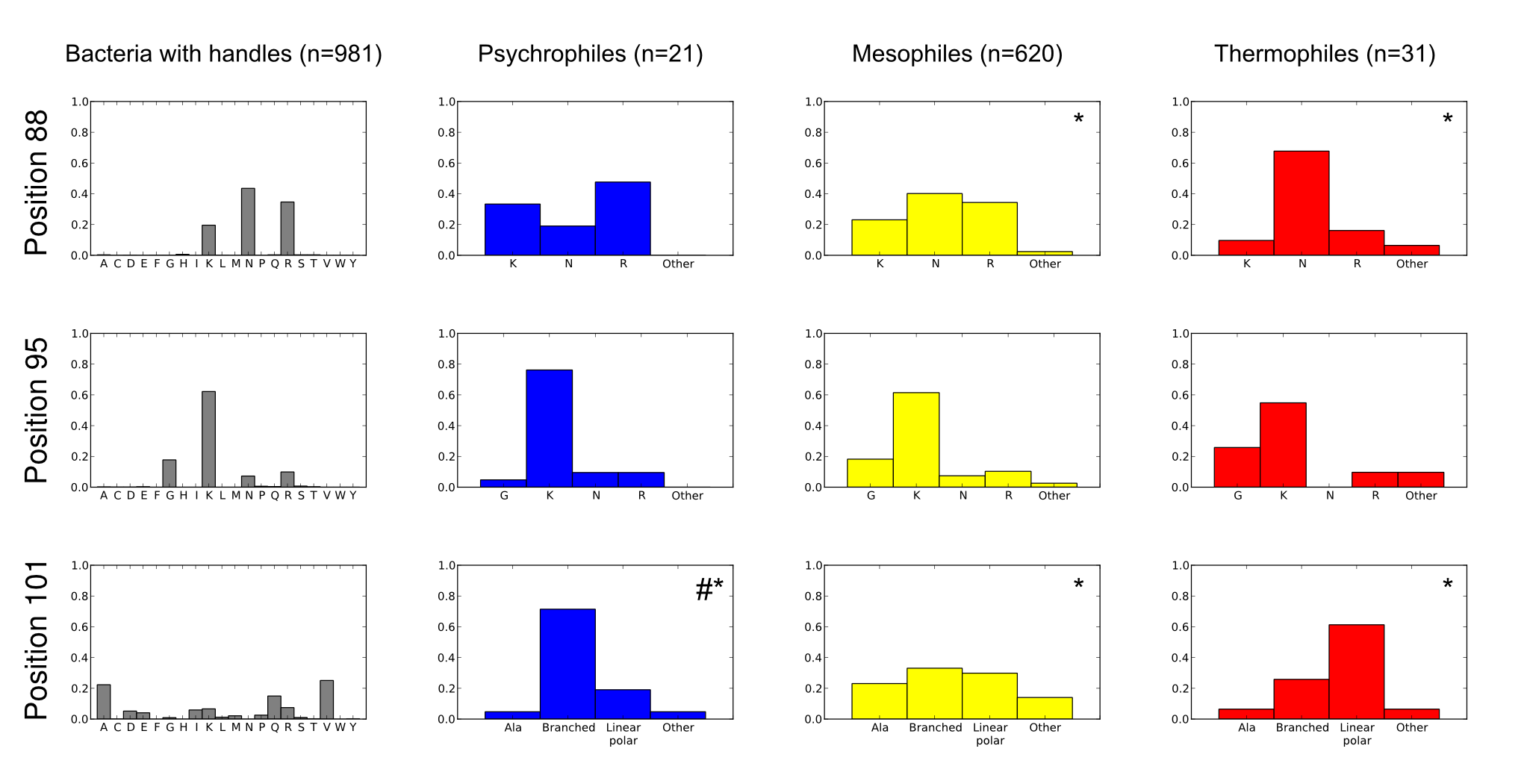

Supplement: Figure S9 — Residue frequencies in sites identified as significant determinants of handle-region dynamics. Distribution of residues at each of positions 88, 95, and 101 among bacterial RNase H sequences from all organisms, and as a function of growth temperature annotation. For position 101, residues have been clustered into four categories: alanines, branched amino acids (isoleucine, leucine, valine), linear and polar amino acids (arginine, lysine, glutamate, glutamine), and other amino acids. For positions 88 and 101, the notation * indicates a distribution significantly different from uniform, and the notation # indicates a distribution significantly different from the overall dataset ( test with Bonferroni-corrected significance level of p<0.003). Mean percent sequence identities for each category are 54% (overall), 62% (psychrophiles), 55% (mesophiles), 51% (thermophiles). (TIFF) [file pcbi.1003218.s009.tiff]
